# *Batavia* shipwreck timbers reveal a key to Dutch success in 17th-century world trade

**DOI:** 10.1371/journal.pone.0259391

**Published:** 2021-10-29

**Authors:** Aoife Daly, Marta Domínguez-Delmás, Wendy van Duivenvoorde

**Affiliations:** 1 Saxo Institute, University of Copenhagen, Copenhagen, Denmark; 2 Dendro.dk, Copenhagen V, Denmark; 3 Department of History of Art, University of Amsterdam, Amsterdam, The Netherlands; 4 Department of Conservation and Science, Rijksmuseum, Amsterdam, The Netherlands; 5 DendroResearch, Wageningen, The Netherlands; 6 College of Humanities, Arts and Social Sciences, Flinders University, Adelaide, Australia; University of Nevada, Reno, UNITED STATES

## Abstract

Ocean-going ships were key to rising maritime economies of the Early Modern period, and understanding how they were built is critical to grasp the challenges faced by shipwrights and merchant seafarers. Shipwreck timbers hold material evidence of the dynamic interplay of wood supplies, craftmanship, and evolving ship designs that helped shape the Early Modern world. Here we present the results of dendroarchaeological research carried out on *Batavia*’s wreck timbers, currently on display at the Western Australian Shipwrecks Museum in Fremantle. Built in Amsterdam in 1628 CE and wrecked on its maiden voyage in June 1629 CE in Western Australian waters, *Batavia* epitomises Dutch East India Company (*Verenigde Oostindische Compagnie*, or VOC) shipbuilding. In the 17th century, the VOC grew to become the first multinational trading enterprise, prompting the rise of the stock market and modern capitalism. Oak (*Quercus* sp.) was the preferred material for shipbuilding in northern and western Europe, and maritime nations struggled to ensure sufficient supplies to meet their needs and sustain their ever-growing mercantile fleets and networks. Our research illustrates the compatibility of dendrochronological studies with musealisation of shipwreck assemblages, and the results demonstrate that the VOC successfully coped with timber shortages in the early 17th century through diversification of timber sources (mainly Baltic region, Lübeck hinterland in northern Germany, and Lower Saxony in northwest Germany), allocation of sourcing regions to specific timber products (hull planks from the Baltic and Lübeck, framing elements from Lower Saxony), and skillful woodworking craftmanship (sapwood was removed from all timber elements). These strategies, combined with an innovative hull design and the use of wind-powered sawmills, allowed the Dutch to produce unprecedented numbers of ocean-going ships for long-distance voyaging and interregional trade in Asia, proving key to their success in 17^th^-century world trade.

## Introduction

The expansion of Dutch seafaring in the Early Modern Period is documented extensively in historical archives, especially regarding the Dutch East India Company (*Verenigde Oostindische Compagnie*, hereafter VOC) [1–6]. Since its foundation in 1602 CE, the VOC pioneered the model for a joint-stock company with a permanent capital base; hence, it is often considered the world’s first globalised enterprise at the dawn of modern capitalism [3–6].

During the 17th century, the VOC led the European trade with and within Asia [1–4], operating a fleet of large ocean-going vessels that were built and fitted in VOC shipyards in the Dutch Republic [7]. Shipbuilding activity thrived despite the lack of native woodlands within the Republic’s confinements and hinterland. Since the late Middle Ages, trade networks supplied imported wood from different sources [6,8], and, by the early 17^th^ century, wood had become one of the five main import products in the Dutch Republic, together with grain, salt, herring and textiles [6]. VOC archives in the Netherlands and abroad retain detailed accounts of the Company’s transactions and activities for the period 1602–1795 CE and represent such an exceptional historical register that they were included in UNESCO’s Memory of the World Register in 2003 [9]. Still, VOC archives from the early 17th century pertaining to timber procurement, provenance, and imports are practically non-existent and its early shipbuilding charters are difficult to translate and interpret, leaving unsolved questions that cannot be answered through the study of archival records alone [7,10]. Fortunately, material evidence from those flourishing times can be found on VOC shipwrecks around the world. Remains of Dutch ships preserved under water represent unique archaeological datasets for the evolution of shipbuilding and global seafaring in the Early Modern Period [11–15]. Dendrochronological analysis of shipwreck timbers provides direct evidence of ancient craftmanship and woodworking techniques, timber procurement areas, and trade connections in specific historical periods [16], bridging the gap between the historical and archaeological records.

## *Batavia*: Epitome of early 17th-century shipbuilding

**Fig 1:**
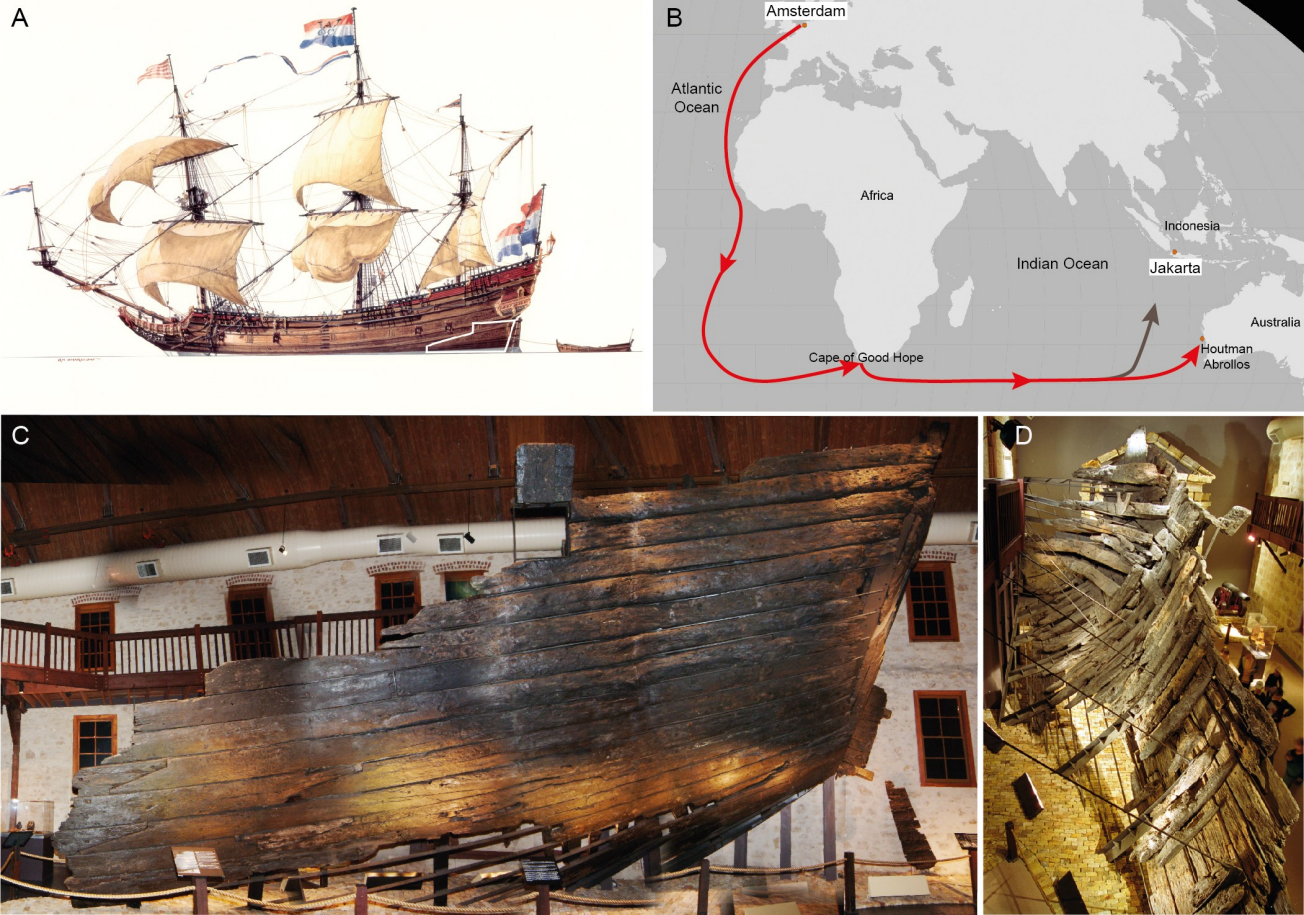
*Batavia* ship and shipwreck. A) Ship illustrated by Ross Shardlow, with a white line indicating the preserved section of the port-side and transom raised from the shipwreck site; B) route sailed from the Netherlands to the wreckage site on Morning Reef off Beacon Island (Houtman Abrolhos Archipelago); C,D) ship’s hull on display at the Western Australian Shipwrecks Museum in Fremantle (Photos: P.E. Baker, Western Australian Museum).

*Batavia* (Fig 1A) is the most emblematic VOC shipwreck found so far. Sometime after the spring of 1626 CE, Jan Rijcksen, Master shipwright of the Company’s Amsterdam shipyard, commenced construction of two new ships—the larger one would be named *Batavia* [7]. *Batavia* measured 160 Amsterdam feet (45.30 m) in length over its upper deck, 36 Amsterdam feet (10.19 m) in beam, and the height between the top of its keel and lower deck was 14 Amsterdam feet (3.94 m). The ship’s length-to-beam ratio was 4.4:1, and its volume measured 300 *lasten* (600 metric tons) [7]. Rijcksen employed an innovative construction method that epitomised VOC shipbuilding in the first half of the 17th century [7]. *Batavia* was constructed using a bottom-based method that applied two thick layers of oak hull planking below the ship’s waterline. This method entailed assembling the bottom hull planking first, before the frames were inserted. Frame timbers function as the internal framework of a ship that strengthen a ship transversely, and they are usually made up of floors (timbers that run over the keel in the bottom hull) and futtocks (build up along the side of the ship). In a bottom-based construction method, the frame floors and first futtocks were fastened to the bottom planking, after which the second futtocks of the ship’s sides were erected [17]. Above the ship’s waterline, hull planks were nailed to these frames in typical plank-on-frame fashion. The method that Rijcksen used to construct *Batavia* was advantageous as it created a ship large and strong enough to withstand the voyage to Southeast Asia and back. It had only been since 1595 that the Dutch commenced sailing to this region and required different types of ships designed and constructed specifically for inter-oceanic travel. Such ships were not commonly available and necessitated new developments from Dutch shipyards [7].

It took Rijcksen almost two and a half years to complete *Batavia*’s construction. At one point, on 25 May 1628, as the ship and its sister were nearing completion, Rijcksen had to travel with two Company administrators to purchase additional timber to complete work on the two vessels [18]. Finally, in October that same year, *Batavia* launched and set sail from Texel, Netherlands, as the flagship in a fleet of six VOC ships bound for Batavia (modern-day Jakarta) in Southeast Asia (Fig 1B). The ship never reached its intended destination. Almost eight months after its departure, on 4 June 1629, *Batavia* ran into what later became known as Morning Reef in the Houtman Abrolhos archipelago, off the western coast of Australia [7,12].

*Batavia*’s shipwreck site was located in the 1960s and excavated in the 1970s. Following a lengthy conservation, its remaining hull structure was reassembled and, since 1991, has featured prominently in the Western Australian Shipwrecks Museum in Fremantle (Fig 1C and 1D).

## A unique resource to study shipbuilding practices

From the time of its departure until its sinking, *Batavia* never underwent repairs or refitting, and so all its timbers belong to the ship’s original construction. Essentially still a brand-new ship when it sank, *Batavia*’s remains provide a unique opportunity and an exceptional resource to study state-of-the-art 17th-century shipbuilding [7].

The archaeological study of *Batavia* hull remains conducted in the early 2010s corroborated historical documents, manifesting that VOC shipwrights prioritised in their construction a strong and watertight hull, well protected from *Teredo* molluscs (shipworms) and other marine organisms [7,19]. They accomplished this by reinforcing the construction in the lower hull with multiple layers of timber elements. Two layers of external oak planking provided the bulk of the hull’s strength, which the builders further reinforced and protected with an outer layer of pine sheathing. They sandwiched the frames between these outer layers and a layer of oak ceiling planking on the interior, which was further strengthened and protected below the lower deck with a pine floor [7,20]. The shipwrights enhanced the hull’s watertightness by slightly offsetting the seams of the two layers of oak hull planking, reminiscent of the way overlapping roof shingles are laid. The builders gave less attention to *Batavia*’s frames, as these simply acted as lateral stiffeners in the hull.

This construction method with multiple layers of ship elements, from the outside to the inside, included one layer of pine sheathing, two layers of hull planking, one layer of frames, and one layer of ceiling planking covered by another layer of pine flooring. This produced an extraordinarily thick hull, which created a laminate-type layering of timbers in the lower hull; the combined thickness of *Batavia*’s two layers of hull planking alone was 18 cm. *Batavia* was not exceptional in this regard, as this multi-layered bottom-based hull construction has been confirmed in the archaeological remains of other VOC ships and yachts and by the Company’s shipbuilding charter from the early 17th century [7]. *Batavia*’s outer layer of pine sheathing, which in places could be as much as 5 cm thick, reinforced the two main layers of hull planking. More importantly, though, it protected them below the waterline from the ravages of marine organisms and, for this reason, is referred to by some as sacrificial planking. It was fastened to the outer layer of hull planking with closely spaced iron nails. As the nails corroded, they created a layer of iron oxide that further helped repel marine organisms [7,20]. All told, the combined thickness of *Batavia*’s outer hull planking was over twice that of contemporary French, English, and Dutch single-planked vessels. Rijcksen and his builders took a similar approach to strengthening and protecting *Batavia*’s sternpost (and, presumably, its stem, although no direct evidence of its treatment is preserved). They covered the sternpost with a layer of oak sheathing, followed by a layer of copper sheets [20,21], and then added a layer of pine sheathing fastened with iron nails in the same manner as the hull.

Since the precise construction date and place of *Batavia* are known, dendroarchaeological analysis of the ship’s timbers would reveal how the VOC organised the supply of timber for its shipbuilding enterprises in the 1620s CE, in terms of supply areas, selection of timbers and timber products, and seasoning times for fresh-cut wood. Such empirical data could then be compared directly to archaeological evidence of the ship’s construction, as well as to VOC archival documentation. Therefore, our research aimed to carry out an extensive and comprehensive tree-ring analysis of *Batavia*’s wooden hull remains to provide a snapshot of VOC shipbuilding practices in Amsterdam in the late 1620s CE.

## Materials and methods

### Material

*Batavia*’s surviving hull section is a portion of the lower port stern and transom preserved to just above the lower deck (Fig 1A, 1C and 1D) and comprises the remains of 21 hull planking strakes, 46 frames and a gun port with lid. No timbers from the ship’s bottom, like the keel and lowest hull planking, nor any timbers from *Batavia*’s starboard side have survived [7].

A total of 137 samples were collected from 101 timbers (conserved by treatment with polyethylene glycol (PEG)) and analysed by dendrochronology between 2007 and 2017 (see detailed sampling history in S1 Appendix and S1 Table). Although 12 samples represent cross-sections of timber elements that were stored in boxes after being conserved, all remaining samples were obtained from timbers in the ship display, collected either with a manually driven Haglöf increment borer of 5 mm diameter, or with a 16 mm diameter dry-wood borer driven by a power-drill.

All necessary permits were obtained for the described study, which complied with all relevant regulations. The sampling was conducted with the approval of the Western Australian Museum (21st January 2017, Specimen Invoices No. A0500 and A0022) and permission to undertake further analysis was granted under General Permit No. 25 which allows the Western Australian Museum to export loan specimens that are subject to the Commonwealth *Protection of Movable Cultural Heritage Act* 1986 and *Historic Shipwrecks Act* 1976. The samples were returned after study and remain in the collection of the Western Australian Museum.

### Tree-ring measuring and dating

To allow the clear visualisation of tree-ring boundaries, radii were cleaned with razorblades on the transverse surface of the cross-sections from the inner to the outermost ring following standard dendroarchaeological procedures [22]. The same approach was employed to clean the surfaces of the cores. When necessary (e.g., to visualise areas of growth reductions), superficial PEG was removed from the surface with a warm wet cloth and chalk powder was applied to enhance the visualisation of tree-ring boundaries. Ring-widths were measured to the nearest 0.01 mm in three different labs with different devices (S1 Appendix). Tree-ring patterns of some samples were recorded in a sequence of overlapping photographs with an automatic camera on macro-lens mode, and ring-widths subsequently were measured on the photographs using the CooRecorder&CDendro software package [23] or AbleImageAnalyzer software [24].

Crossdating was done in a three-step approach that followed standard procedures [22,25]. First, the tree-ring series of the individual samples were cross-compared to identify timbers potentially cut from the same tree (S2 Table). These were averaged into series representing individual trees, which again were cross-compared to find series with high agreement. These were grouped and averaged into object chronologies that represent timbers deriving from trees that grew under similar conditions, likely in the same area (S3 Table). The object chronologies and remaining individual series were compared with site and regional reference chronologies from central and northern Europe to identify their date and provenance, and finally these results were mapped according to the technique described by [26].

### Estimation of felling dates

None of the samples contained sapwood (outermost part of the wood in the stem and branches of trees, distinguishable in oak by its lighter colour even in PEG-treated wood, and usually, showing vessels free of tyloses). Therefore *post quem* dates were estimated for the felling of trees, while *ante quem* dates are the construction years 1626–1628 CE, based on the VOC archives [7]. Such estimations of felling dates consider the minimum number of sapwood rings that may be missing towards the outside of a tree until the bark. Observations about the number of sapwood rings in living trees have been compiled for different regions in Europe and have been found to decrease from west to east [27]. Thus, the provenance of the timber must be known to implement sapwood estimates corresponding to the region of origin. Here we used the estimations compiled by [27], which include the ones proposed by [28] for the Baltic area (which add nine rings to the end date in case of missing sapwood), and the ones proposed by [29] for Germany, which add 12 rings to the end date of timbers derived from trees suspected to be younger than 100 years, and 14 rings to those derived from trees between 100 and 200 years of age. For timbers with an undefined provenance, we considered also the estimation by [29].

## Results

### Dendrochronological dating

Samples were taken from a total of 101 ship timbers comprising 98 framing elements and hull planks of deciduous oak (*Quercus* subg. *Quercus*) and four sacrificial planks of pine (likely *Pinus* sylvestris) (S1 Appendix and S1 Table). Dendrochronological research resulted in the absolute dating of 53 (c. 54%) oak timbers, whereas the pine samples remain undated. The 45 undated oak samples originated from planks (N = 27) or frames (N = 18) with wide and few rings (most had less than 60 rings; Fig A in S2 Appendix, and S1 Table). In the case of the planks this resulted from a combination of sampling at a shallow angle tangential planks that were likely obtained from the inner part of the tree, where tree rings can be wider. Therefore, those samples do not necessarily reflect the growth rate or the age of the tree and were left out of further analysis.

**Fig 2:**
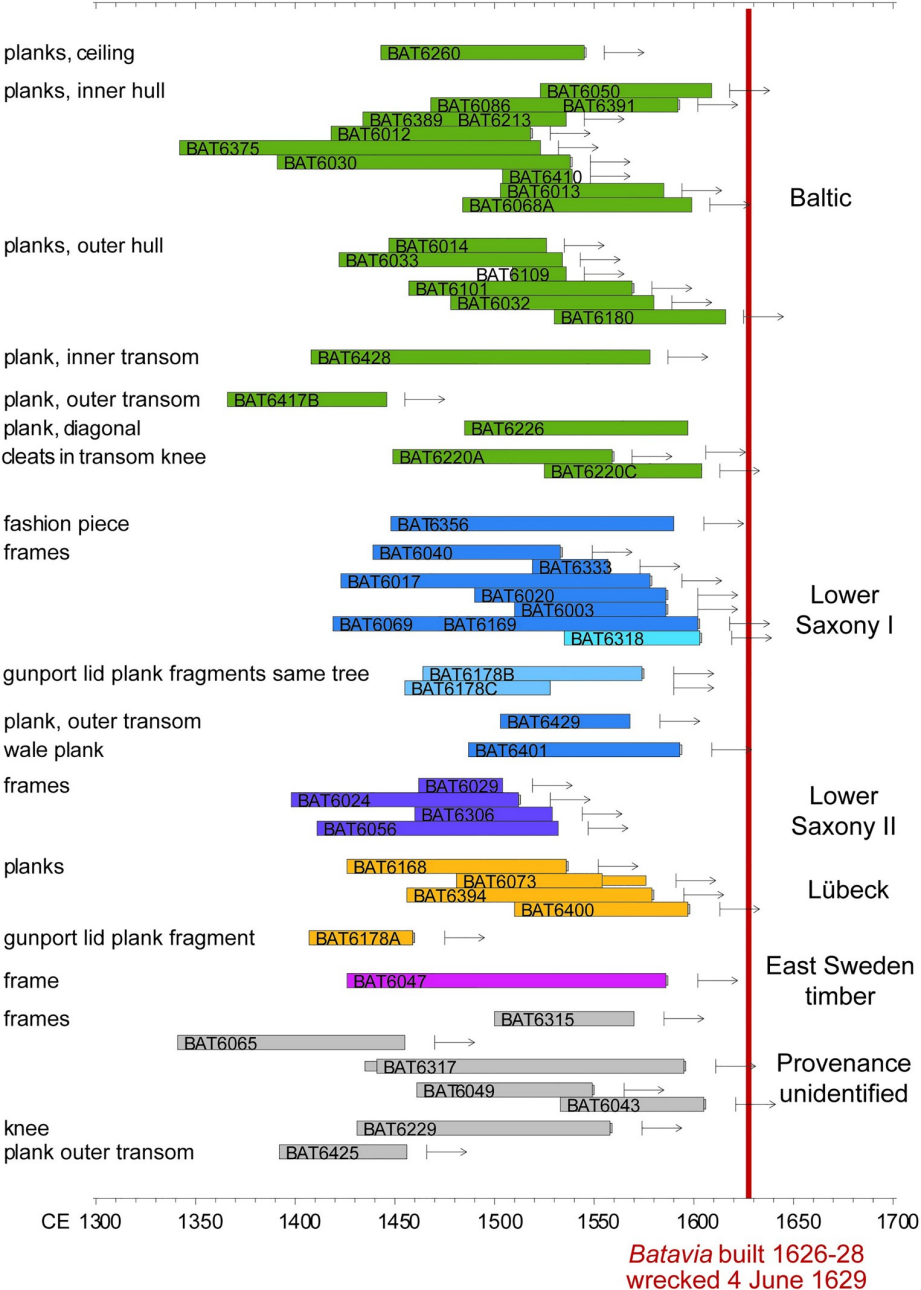
Dendrochronological results. Each bar represents the time span covered by the dated timbers. The bars are coloured according to the dendrochronological groups that have been identified. The red vertical line marks the dating of the building and wrecking of *Batavia*.

The outermost tree ring among the dated frame samples was formed in 1605 CE, and that of the dated hull planks in 1616 CE (Fig 2). The estimated felling dates for both trees can be extrapolated to 1625 or later (having allowed for missing sapwood), which is consistent with the historical dating for the ship’s construction between 1626 and 1628 CE [7]. Several other timbers also have outer rings dating in the 1600s CE. It is thus probable that the timber was processed shortly after felling of the trees, as logs were easier cut into smaller sections and planks could be bent to shape during the ship’s construction while still green [7].

### Three main provenance groups

Three main groups and one smaller group of timbers displaying high similarity were identified corresponding to trees that grew under similar conditions (S1 Appendix). Tree-ring series of some of the inner hull planks and futtocks displayed such visual and statistical similarity that it was possible to establish or confirm that they belonged to the same ship timber (broken or sawn when the hull was raised in the 1970s) (S2 Table), resolving questions that had emerged during the archaeological recording of the ship’s remains about the possibility that different fragments of wood corresponded to the same timbers [7].

**Fig 3:**
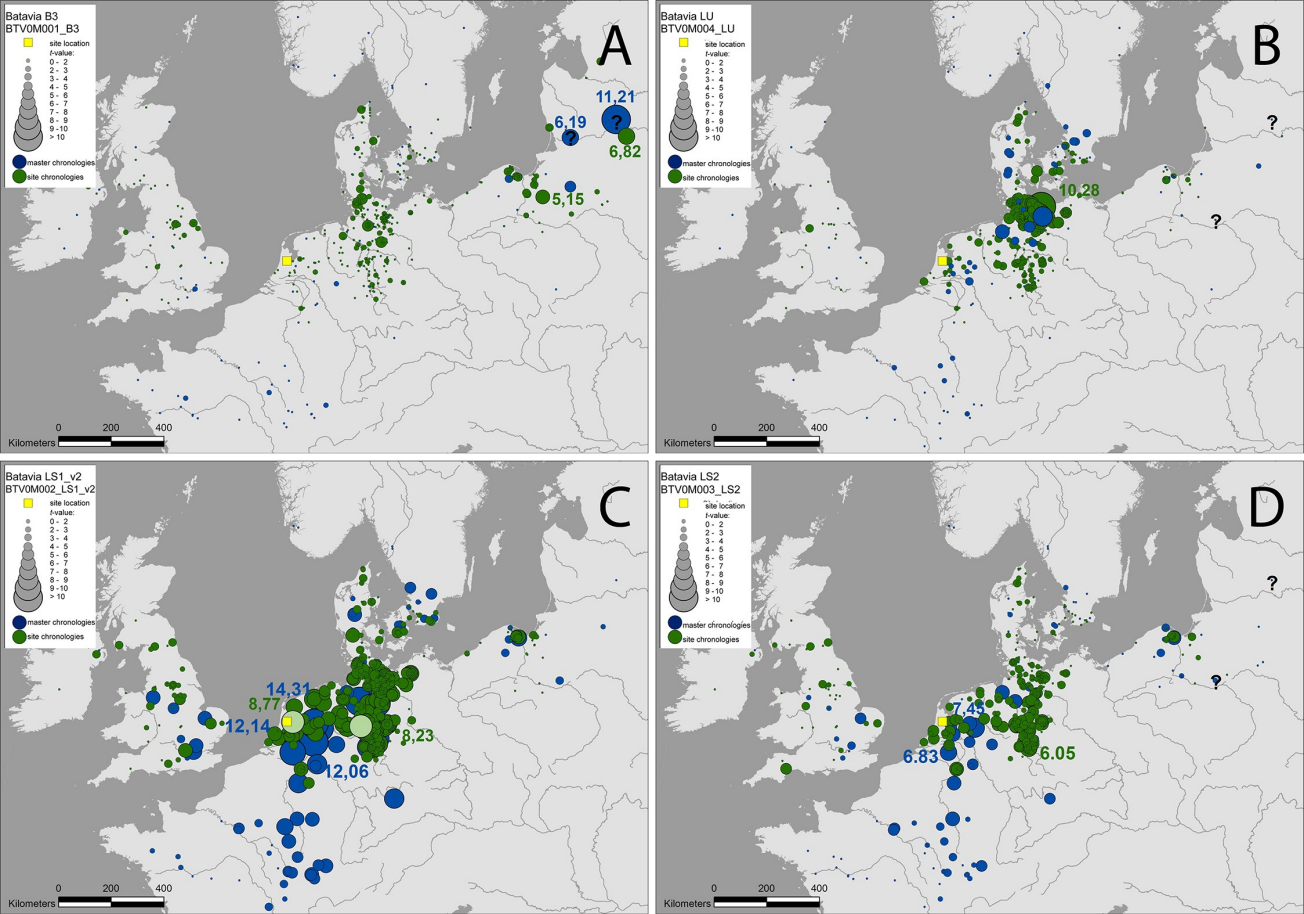
Timber provenance maps. Each dot represents the location of a site chronology (green) or a regional chronology (blue). The *t*-value is the correlation measure used here (adapted for dendrochronological studies by [31], and each circle represents the *t*-value achieved with each dataset—the larger the circle, the higher the *t*-value. (For a detailed explanation of the technique used here and the underlying tree-ring data see [26]). A) Baltic Group; B) Lübeck Group; C) Lower Saxony I Group; D) Lower Saxony II Group.

The three main groups correspond to diverse regions of origin (Figs 2 and 3): south-eastern Baltic (Baltic Group), northern Germany (Lübeck Group), and north-western Germany (Lower Saxony Group). The Baltic Group comprises 20 timbers represented by 22 tree-ring series. These were averaged into an object chronology covering 275 years, from 1342 to 1616 CE (S3 Table). The highest correlations appear with reference chronologies from the southern and eastern Baltic region and with art-historical chronologies made in western Europe from oak used in fine-art panel paintings [30] (Fig 3A). All the timbers included in this group are planks (inner and outer hull planks below the waterline, ceiling and transom planks). Two small planks forming a cleat at one of the transom knees also belong to the Baltic timber group. All these planks were tangentially converted from the parent tree.

The Lübeck Group consists of five timbers (five tree-ring series) that have been combined into an object chronology covering 191 years, from 1407 to 1597 CE (S3 Table). This average shows the highest correspondence with site chronologies in the vicinity of Lübeck (Fig 3B). All timbers in this group are inner hull planks above the waterline, except one small plank piece used to complete a gunport lid.

The Lower Saxony Group is divided into two sub-groups (I and II) (Fig 3C, S3 Table). Lower Saxony Group I consists of 13 timbers representing 11 trees (two pairs of timbers come from the same tree), and their object chronology (built with the 10 best-matching tree-ring series) covers 184 years, from 1419 to 1602 CE. The geographical correlations (*t*-value [31]) with master and site chronologies extend from Lower Saxony to the Netherlands, but the best match is achieved with a chronology representing the historical area of Twente/Westphalia in the eastern Netherlands and north-western Germany. Most timbers in this group are frames, positioned throughout the reassembled ship remains, but others were used as transom and hull planking above the ship’s double planked bottom. Two short fragments of one timber served to complete the aforementioned gunport lid. Lastly, four timbers from futtocks in *Batavia*’s framing structure are assigned to Lower Saxony Group II and, their four tree-ring series have been averaged into an object chronology covering 135 years, from 1398 to 1532 CE. The highest correlations are with chronologies in Lower Saxony and The Netherlands, and the best agreement again is with the chronology for Twente/Westphalia.

Seven dated timbers cannot be assigned to any of the groups (Fig 3D). These timbers date with diverse master and site chronologies, but the correlation values achieved are insufficient for precise identification of their provenance.

One frame timber has a different provenance than the rest of the framing timbers from the *Batavia* hull and matches best with chronologies derived from timbers of two other shipwrecks. One of these was found in 2017 at Skeppsholmen, in Stockholm, and has been identified as the remains of the Swedish-built ship *Sceptre* (1615 CE) [32–34]. The other one is the warship *Vasa*, built in 1628 in Stockholm, which also sunk like *Batavia* on its maiden voyage, in Stockholm Harbour [35,36]. Therefore, the outlying *Batavia* frame timber is likely originating from a forest in eastern Sweden.

### Differentiated supply of timber products

**Fig 4:**
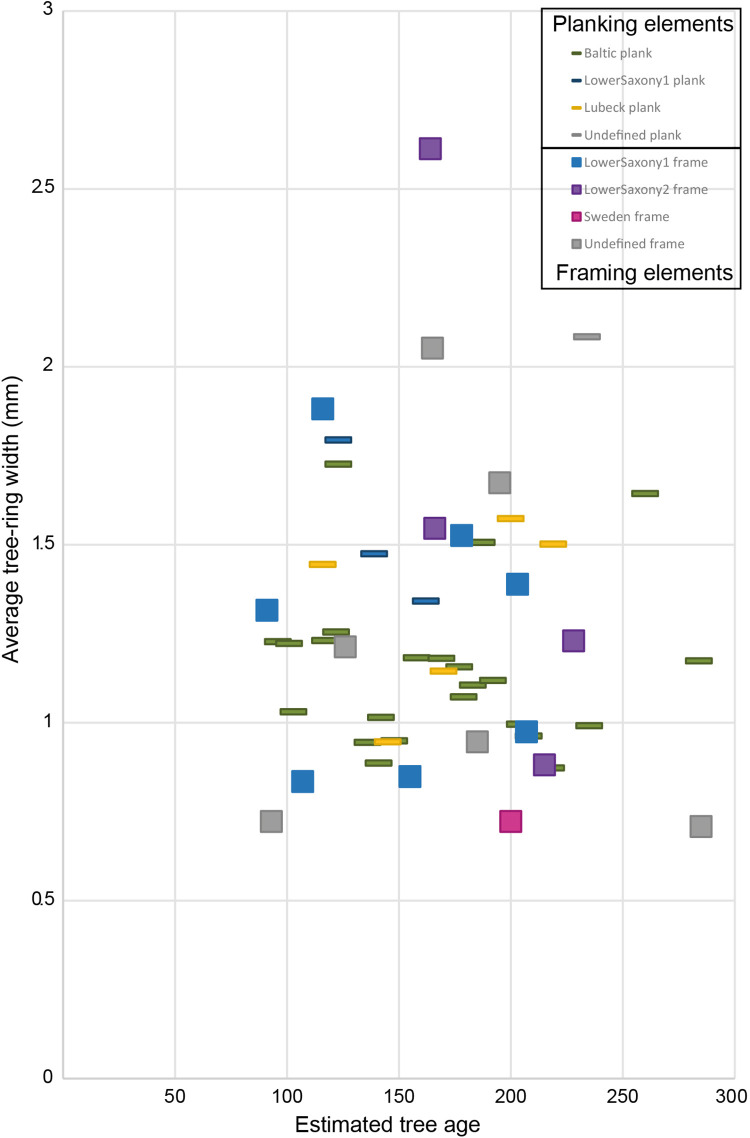
Growth rates (mean ring width) and tree ages per provenance and type of timber. Growth rates of framing and planking elements vary similarly (hence no discriminant selection of trees has been made for specific timber elements), but timber elements show a distinct discrimination based on geographical area (framing elements were obtained primarily from the Lower Saxony in north-west Germany, while planks were mainly obtained from the Baltic and Lübeck area). The tree ages have been estimated considering that all the trees from the dated timbers were cut in 1625. Since the pith is absent in all the samples, some trees might be older than estimated.

The results of this research show that trees sourced in each provenance group have similar average growth (Fig 4; S2 Appendix), although part of the undated samples (the framing elements) may represent younger and faster growing trees. Generally, this indicates that timber selection for hull elements (planks and frames) was not based on differentiated growth rates (e.g. slow-grown trees selected for planks and fast-grown trees selected for framing elements, as was the case for 15th century shipbuilding elsewhere [37–39]). Instead, the picture that emerges is that VOC shipwrights sourced specific timber products from specific regions to make particular structural elements (Fig 4): while wood for planking elements was primarily sourced in the Baltic and Lübeck area, timber for framing elements was sourced in Lower Saxony (northwest Germany).

**Fig 5:**
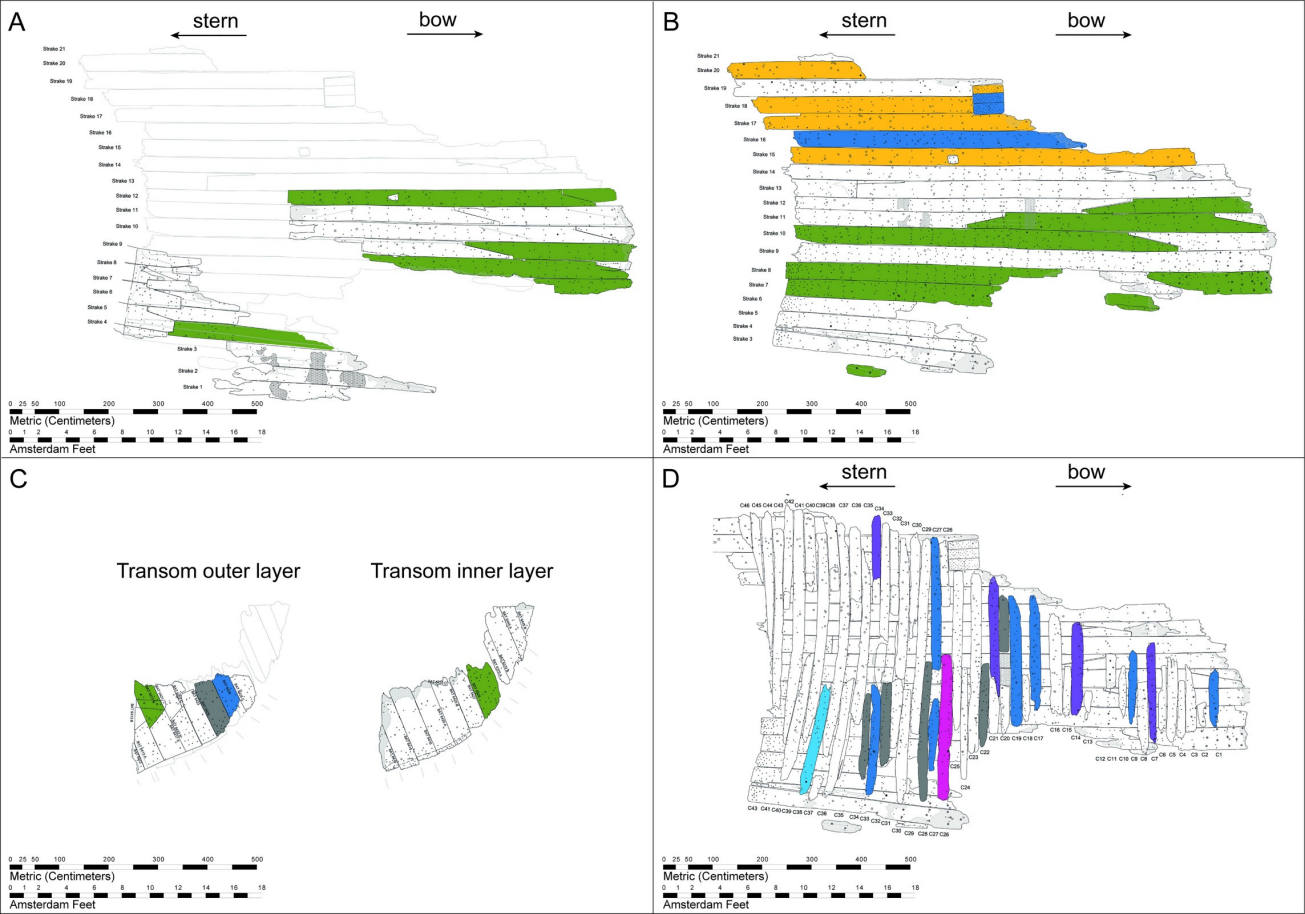
Distribution of the dated timbers in some parts of the shipwreck, coloured by provenance. A) Inner hull planks; B) Outer hull planks; C) Transom planks; D) Framing elements.

Furthermore, we have observed a distinct disposition of the timber provenance in the shipwreck (Fig 5). All of the planking timber used in the lower hull (internal and external) was sourced from the east Baltic region while timber for the ship’s upper hull planking (above the waterline) is predominantly from east of the Øresund (Danish straits) around Lübeck. The framing elements (frames, futtocks, lodging knees) came from regions further west; Lower Saxony, possibly also Scandinavia, were the favoured sources framing timbers. We thus see a highly differentiated source for planking versus bulk timber in the ship remains.

## Discussion

### Innovative ship design

Archaeological studies have confirmed that, from the later years of the 16th century CE into the second half of the 17th, all Dutch ships designed for the trade with Asia were outfitted with double-planked hulls [7,15,20,21]. Unlike Dutch flutes and other bulk cargo-carriers intended for deployment in European waters, the East India ships of Dutch joint-stock companies and the VOC were much more robustly built than previously thought. Stronger hulls were necessary to meet the challenges of the longest and most distant voyaging of the VOC and, in the process, protect cargoes, lives, and investments. The superior hull integrity of VOC ships was owed to the early and innovative application of multiple layers of timber. Dutch shipwrights were still using a bottom-based approach, in which a ship’s hull integrity relied predominantly on a sturdy shell of planking, but to strengthen this further, they divided the thicker hull skin into two layers so as to facilitate the bending of heavy oak planks and keep them in place. Two thinner layers of hull planking were probably also more resilient than one very thick layer and were easier to repair [7]. This type of construction is better known from whaling ships, but the earliest historical references for reinforcing Dutch whalers with an additional layer of oak planking date only to 1660, coinciding with the beginning of Dutch fishing and whaling in the icy waters around Spitsbergen [21]. The double-planking of whaling ships thus seems to have commenced more than 50 years after the VOC had started the practice in the construction of its East India ships.

Such innovative ship design required high amounts of timber, which the VOC managed to obtain through a well-established trade network [6]. Dendrochronological studies on archaeological structures and on furniture and works of art produced in the Netherlands in the 16th and 17th centuries have attested the flow of oak timber into the former Dutch Republic. This timber originated largely from the Baltic (mainly used in works of art and furniture up to c. 1650) and different parts of Germany (used in buildings, infrastructures, and from c. 1650 onwards, on works of art) [40–42]. Our results, albeit derived from a small portion of the ship’s hull, contribute to the collective knowledge about north European timber trade and illustrate the geographical extent of areas supplying timber for shipbuilding in the Dutch Republic in the 17th century.

### Skilled craftmanship

The end dates of the timbers, between the late 1590s and the 1610s (Fig 2), suggest that only the sapwood was removed when the felled trees were converted into the final ship timbers. Such practice reflects a profound knowledge about the soft and perishable nature of sapwood (the outermost part of the wood, just beneath the bark), and its susceptibility to insect attack. Although we cannot dismiss outright the possibility that the sapwood of at least some timbers may have been lost during recovery and/or conservation, it is highly unlikely that this happened to all of them. Well preserved sapwood often is found in oak ship timbers that have been under water for centuries [43–50]. Framing elements sometimes retain even the terminal ring under the bark, providing the exact year in which the trees were cut [49,50]. Therefore, the removal of sapwood must have occurred at the shipyard, after transport, while crafting the timbers into their final form. This not only demonstrates high-quality woodworking standards, but also an intentional pursuit of a robust and durable ocean-going sailing vessel.

### Masterly timber selection

The preference for specific timber products from selected regions demonstrates that the choice of timber was far from arbitrary. Baltic oak was renowned for its exceptional quality [51]. Baltic woodlands produced slow-growth trees with straight-grained trunks. Also known as art-historical oak, straight-grained trunks were commonly used in western Europe up to the middle of the 17th century to make panels for fine-art paintings [52]. VOC shipwrights prized this timber for the bottoms of their hulls, as from it they could fashion sturdy, thick, and long planks. Trees in Lower Saxony must have grown in a different type of environment, as suggested by the crooked and knotty shape of the framing elements. The possibility that the frames derived from branches cannot be discarded. This not only demonstrates the deep knowledge of VOC shipwrights of the timber products available from different forest regions and their specialized use for different purposes, but it also reveals how they perceived and designed the hulls of their ships, with a double-plank bottom constructed from the best material. The various framing elements were secondary and played predominantly a reinforcing role, as reflected in *Batavia*’s framing elements. They were of a lesser quality, sourced closer to home (Lower Saxony in northwest Germany), presumably at lower costs [53].

### A key to Dutch success in world-wide trade

Among the factors to which historians have ascribed the success of the Dutch economy in the 17th century, entrepreneurship, an early (wind-powered) industrial revolution, and diversification of international trade feature prominently [54,55]. These factors were made possible, whether directly or indirectly, by ships like *Batavia* [3,5,6]. Shipbuilding thus was paramount to Dutch economic supremacy in the 17th century, but, in turn, was also advantaged by industrial and economic innovation and the adoption and proliferation of wind-powered sawmills. The financial innovation of joint stock companies provided the capital necessary for global-scale trading ventures. Innovative construction methods supported by specialized procurement, processing, and allocation of shipbuilding timber produced the most efficient and robust ocean-going vessels of the day. Our results demonstrate that the Dutch were able to cope with their ever-growing demand for ship timber by diversifying their sources and integrating them into long-standing trade connections; this at a time when other maritime nations, such as Portugal, Spain, France, and England, were struggling to supply their navies [56–59].

From a scale standpoint, the widespread utilization of wind-powered sawmills marked a true revolution for the Dutch shipbuilding industry [60]. These sawmills could process 60 beams in 4–5 days, whereas hand-sawing the same amount of timber took 120 days [7,61]. This technological innovation and its close integration with supply chains and shipyards enabled the Dutch to construct hundreds of ocean-going vessels in the early 17th century, in the process consuming annually nearly 320,000 m^3^ of oak [7,61]. In Holland, the first sawmill was built in 1594 by Cornelis Corneliszoon, a hand sawyer and millwright from Uitgeest [60]. Such mills proliferated rapidly in Amsterdam province and especially in the Zaan district (Zaanstreek) of North Holland, which developed into a massive industrial complex dedicated largely to shipbuilding and outfitting [6,62,63]. Nevertheless, the *Batavia* ship was built exclusively with manually sawn timber, as the hand-sawyers’ guilds had managed to keep mechanized sawmills from being established in Amsterdam city [7,64]. Sawmills only began to be built and operated systematically there after 1630, barely two years after *Batavia*’s completion, when the newly established Sawmill Development Company secured a patent [7]. The VOC quickly became one of its most prominent clients [64].

### Dendrochronological research and museum display

The *Batavia* shipwreck has provided the only wooden remains of an early 17th-century VOC ship to be raised from the seabed and conserved to permit detailed study. This is a most exceptional research object, given that i) partial archival resources exist about its construction, ii) it sunk on its maiden voyage without undergoing repairs, iii) the good state of conservation of its timbers and the current display allowed for a thorough sampling of different timber elements for dendrochronological research. Our results illustrate the variety of timber sources supplying the VOC Amsterdam shipyard in the 1620s and demonstrate the builders’ careful timber selection and skilled craftsmanship, insights made possible only through extensive sampling of hull timbers of different sizes, shapes and functions.

Dendrochronological research of shipwrecks usually requires invasive sampling. This is a tedious procedure while the wreck is under water, as the visibility, the accessibility to inspect and sample timber elements and the environment in which the wreck is found (depth, sediment, etc.) pose challenges that may hinder greatly the number of samples that can be taken [16]. In the case of recovered shipwrecks that are going to be displayed, sampling is typically deemed incompatible with musealisation. Several examples, however, now disprove this notion. Studies of the ship remains at the Viking Ship Museum in Roskilde, Denmark [65]; the Bremen cog (1378) at the German Maritime Museum [66]; *Vasa* (built 1626–1628), at the eponymous museum in Stockholm, Sweden [35]; *Mary Rose* (built 1510/11), on display at the Mary Rose Museum in England [67]; and now *Batavia*, at the Western Australian Shipwrecks Museum, all testify to the compatibility of tree-ring investigations with musealisation. Respectful sampling, facilitated by close collaboration between conservators and archaeologists, allows for a sensitive sampling strategy tailored to each specific shipwreck, its integrity, and its display. The valuable insights obtained from research on these recovered assemblages should encourage further collaborative projects to maximize the outcomes of studies on extant shipwrecks.

## Supporting information

S1 Table List of timbers sampled and dating results. (PDF)

S2 Table Timbers originating from the same tree. (PDF)

S3 Table Object chronologies. (PDF)

S1 Appendix Sampling history. (PDF)

S2 Appendix *Batavia* timbers–forests of origin and selection of trees. (PDF)
