# Supplementary material for: Batavia shipwreck timbers reveal a key to Dutch success in 17th-century world trade
Source: PLoS One. 2021 Oct 29;16(10):e0259391. doi: 10.1371/journal.pone.0259391 (PMC8555829; doi:10.1371/journal.pone.0259391)
Supplement: S1 Table — (PDF) [file pone.0259391.s001.pdf]

S1 Table. List of timbers sampled and dating results. N: number of rings; SW: number of sapwood rings in the sample; MRW: mean ring width (mm).

Timber code / element / Species	Dendro-code	N	start yr.	end yr.	pith	SW	bark?	Con-version*	extra end	MRW mm	Interpret./ felling
**Samples 2007 – cross-sections**											
BAT6375 (Hanraets DCCD) QUSP	ABA00011	182	1342	1523	?	0	N	?	N	1.17	after 1532 B3 group
BAT6226 (Hanraets DCCD) QUSP	ABA00021	113	1485	1597	?	0	N	?	N	0.89	after 1606 B3 group
BAT6068A (Hanraets DCCD) QUSP<br>BAT6068B QUSP	ABA00032<br>aba00041	116<br>95	1484<br>1498	1599<br>1592	?<br>?	0<br>0	N<br>N	?<br>?	N<br>N	1.01<br>0.88	after 1608 B3 group
BAT6180 QUSP	aba00050	87	1530	1616	?	0	N	?	N	1.23	after 1625 B3 group
BAT6441 PISP	aba00060<br>aba00110	116			?	0	U	?	N	1.35	Undated
BAT No Number PISP is this BAT6150?	aba00100	49			?	0	U	?	N	1.96	Undated
**Samples 2012 – cross-sections**											
BAT6050 QUSP	aba00080	87	1523	1609	?	0	N	?	N	1.03	after 1618 B3 group
BAT6452 from photo QUSP	BAT6452_7b	105			?	0	U	T	H1	1.65	Undated
BAT6470 PISP	aba00130	87			?	0	U	?	N	1.71	Undated
BAT6088 PISP	BAT6088_129	252			G	0	N	T	N	0.81	Undated
BAT6101 QUSP	BAT6101_9a	113	1457	1569	G	0	N	T	H1	1.18	after 1579 B3 group
**Samples 2015 – 5 mm cores**											
6014 hull plank outer layer QUSP	BAT6014a	93			G	0	N	T	H1	0.99	Undated sampled again in 2017
6020 frame QUSP	BAT60208	97	1490	1586	G	0	N	?	H1	0.94	after 1602 LS1 group
6030 hull plank inner layer QUSP	BAT6030b	148	1391	1538	G	0	N	?	H1	0.99	after 1548 B3 group
6031 hull plank inner layer QUSP	BAT6031a	34			G	0	N	T	H1	1.07	Undated
6040 frame QUSP	BAT6040a	95	1439	1533	G	0	N	O	H1	1.51	after 1549 LS1 group
6047 frame QUSP	BAT6047a	161	1426	1586	G	0	N	O	H1	0.72	after 1602 Swedish?
6073 hull plank QUSP	BAT60739	74	1481	1554	G	0	N	?	H22	0.95	after 1591 LU group
6086B hull plank inner layer QUSP	BAT6086a	125	1468	1592	G	0	N	T	H1	1.18	after 1602 B3 group
6168 hull plank QUSP	BAT61689	111	1426	1536	G	0	N	T	H1	1.57	after 1552 LU group
6178A gunport lid top plank QUSP	BAT6178Aa	53	1407	1459	G	0	N	T	H1	1.50	after 1475 LU group
6178B gunport lid lower plank QUSP	BAT6178Ba	111	1464	1574	G	0	N	T	H1	1.34	after 1590 LS1 group
6178C gunport lid wale plank QUSP	BAT6178Ca	74	1455	1528	G	0	N	T	N	1.69	after 1590 LS1 group
6213 hull plank inner QUSP	BAT6213a	72	1452	1523	G	0	N	T	H5	1.28	after 1545 B3 group
6333 frame QUSP	BAT6333a	43			G	0	N	O	N	1.12	Undated sampled again in 2017
6391 hull plank inner layer QUSP	BAT6391a	57	1531	1587	G	0	N	T	H1	1.30	after 1602 B3 group
6394 hull plank QUSP	BAT6394a	124	1456	1579	G	0	N	T	H1	1.15	after 1595 LU group
6400 hull plank QUSP	BAT64009	88	1510	1597	G	0	N	T	H1	1.44	after 1613 LU group
6401 hull plank second wale QUSP	BAT6401a	107	1487	1593	G	0	N	?	H1	1.48	after 1609 LS1 group
**Samples 2017 – 7 mm cores**											
BAT6417A Transom planking outer layer QUSP	BTV0001A	25			?	0	N	?	N	2.64	Undated
BAT6421 transom plank outer layer QUSP	BTV00029	104			G	0	N	T	H1	1.48	Undated
BAT6024 frame QUSP	BTV00033_9	115	1398	1512	G	0	N	?	H1	1.23	after 1528 LS2 group
BAT6434 Cover planking QUSP	BTV0003A1<br>BTV0003A2	14<br>27			?<br>?	0<br>0	N<br>N	?<br>?	N<br>N	1.89<br>1.96	Undated
BAT6367 hull plank inner layer QUSP	BTV00040	87			?	0	N	?	N	1.43	Undated
BAT6220A cleat at transom knee QUSP	BTV00046_a	111	1449	1559	G	0	N	?	H1	1.16	after 1569 B3 group
BAT6260 ceiling plank QUSP	BTV00048_a	103	1443	1545	G	0	N	?	H1	1.11	after 1555 B3 group
BAT6223A transom knee QUSP	BTV00049_a	45			G	0	N	?	H1	1.71	Undated
BAT6296 transom knee QUSP	BTV00050_9v1	73			G	0	N	?	N	1.21	Undated
BAT6261 ceiling plank strake 11 QUSP	BTV00051_a	35			G	0	N	?	H1	2.79	Undated
BAT6218 margin plank QUSP	BTV00052_a<br>BTV00052_b	26<br>24			G<br>G	0<br>0	N<br>N	?<br>?	N<br>N	1.26<br>1.35	Undated
BAT6227 hanging knee QUSP	BTV00053_a	36			G	0	N	?	H1	1.72	Undated
BAT6069 frame QUSP	BTV00054_9	184	1419	1602	G	0	N	?	H1	0.97	after 1618 LS1 group
BAT6003 frame QUSP	BTV00055_a	77	1510	1586	G	0	N	?	H1	1.88	after 1602 LS1 group
BAT6035 frame QUSP	BTV00056_a	11			G	0	N	?	H1	3.91	Undated
BAT6027 frame QUSP	BTV00057_a	44			G	0	N	?	H1	2.99	Undated
BAT6012 hull inner layer QUSP	BTV00058_a	101	1418	1518	G	0	N	?	H1	0.96	after 1528 B3 group
BAT6209 inner plank QUSP	BTV00059_a	52			G	0	N	?	H1	1.45	Undated
BAT6418 Hull planking outer layer QUSP	BTV0005A	68			?	0	N	?	N	1.41	Undated
BAT6017 frame QUSP	BTV00060_a	156	1423	1578	G	0	N	?	H1	1.39	after 1594 LS1 group
BAT6043 frame QUSP	BTV00061_a	73	1533	1605	G	0	N	?	H1	2.05	after 1621 ungrouped
BAT6045 frame QUSP	BTV00062_9	40			G	0	N	?	H1	2.30	Undated
BAT6181 frame QUSP	BTV00063_a	36			G	0	N	?	N	4.10	Undated
BAT6167 frame QUSP	BTV00064_a	61			G	0	N	?	N	2.55	Undated
BAT6337 frame QUSP	BTV00065_a	42			G	0	N	?	H1	4.25	Undated
BAT6169 frame QUSP	BTV00066_a	124	1471	1594	G	0	N	?	H1	0.85	after 1618 LS1 group
BAT6049 frame QUSP	BTV00067_ax	89	1461	1549	G	0	N	?	H31	1.21	after 1595 ungrouped
BAT6333 frame QUSP	BTV00068_a	39	1519	1557	G	0	N	?	H1	0.83	after 1573 LS1 group
BAT6315 frame QUSP	BTV00069_a	71	1500	1570	G	0	N	?	N	0.95	after 1585 ungrouped
BAT6417B Transom plank QUSP	BTV0006A	81	1366	1446	G	0	N	?	N	1.64	after 1455 B3 group
WA BAT6022A frame QUSP	BTV00070_a	38			G	0	N	?	H1	2.99	Undated

Timber code / element / Species	Dendro-code	N	start yr.	end yr.	pith	SW	bark?	Con-version*	extra end	MRW mm	Interpret./ felling
BAT6317 frame QUSP	BTV00071_9	155	1441	1595	G	0	N	?	H1	0.71	after 1611 ungrouped
BAT6318 frame QUSP	BTV00072_a	69	1535	1603	G	0	N	?	H1	1.32	after 1619 LS1 group
BAT6316 frame QUSP	BTV00073_a	17			G	0	N	?	H1	3.44	Undated
BAT6249 Cover planking QUSP	BTV00070	39			?	0	N	?	N	1.70	Undated
BAT6250 Cover planking QUSP	BTV0008A	57			?	0	N	?	N	1.74	Undated
BAT6184 Hull planking inner layer QUSP	BTV0009A	33			?	0	N	?	N	0.99	Undated
BAT6195 Hull planking inner layer QUSP	BTV0010A	22			?	0	N	?	N	1.48	Undated
BAT6388 Hull planking inner layer QUSP	BTV0011A	20			?	0	N	?	N	1.02	Undated
BAT6389 Hull plank inner layer QUSP	BTV0012A	103	1434	1536	?	0	N	?	N	1.12	after 1545 B3 group
BAT6410 Hull plank inner layer QUSP	BTV0013A	36	1504	1539	?	0	N	?	N	1.25	after 1548 B3 group
BAT6102 Hull planking outer layer QUSP	BTV0014A	57			?	0	N	?	N	1.55	Undated
BAT6099 Hull planking outer layer QUSP	BTV0015A	17			?	0	N	?	N	1.82	Undated
BAT6011 Hull planking inner layer QUSP	BAT6011_9	79			?	0	N	?	N	1.99	Undated
BAT6013 Hull plank inner layer QUSP	BTV0017A	83	1503	1585	G	0	N	?	N	1.73	after 1594 B3 group
BAT6029 Frame timber QUSP	BTV0018A	43	1462	1504	?	0	N	?	N	2.61	after 1519 LS2 group
BAT6387 Hull planking, third wale QUSP	BTV0019B	55			?	0	N	?	N	2.10	Undated
BAT6306 Frame timber QUSP	BTV0020A	70	1460	1529	?	0	N	?	N	1.55	after 1544 LS2 group
BAT6308 Frame timber QUSP	BTV0021A	33			?	0	N	?	N	3.44	Undated
BAT6338 Frame timber QUSP	BTV0022A	21			?	0	N	?	N	1.62	Undated
BAT6216 Hull planking QUSP	BTV00230	53			?	0	N	?	N	2.14	Undated
BAT6215 Hull planking first wale QUSP	BTV0024A1<br>BTV0024A2	35<br>58			?<br>?	0<br>0	N<br>N	?<br>?	N<br>N	1.58<br>0.99	Undated
BAT6103 Hull planking outer layer QUSP	BTV00250	65			?	0	N	?	N	1.25	Undated
BAT6033 Hull plank outer layer QUSP	BTV0026A	113	1422	1534	?	0	N	?	N	1.00	after 1543 B3 group
BAT6032 Hull plank outer layer QUSP	BTV0027A	103	1478	1580	?	0	N	?	N	0.95	after 1589 B3 group
BAT6014 Hull plank outer layer QUSP	BTV0028A	80	1447	1526	?	0	N	?	N	1.07	after 1535 B3 group
BAT6106 Hull planking outer layer QUSP	BTV0029A	25			?	0	N	?	N	0.68	Undated
BAT6109 Hull plank outer layer QUSP	BTV0029B short	28	1509	1536	?	0	N	?	N	1.23	after 1545 B3 group
BAT6097 Hull planking inner layer QUSP	BTV0030A	30			?	0	N	?	N	2.20	Undated
BAT6065 Frame timber QUSP	BTV0031A	115	1341	1455	?	0	N	?	N	1.67	after 1470 ungrouped
BAT6058 Frame timber QUSP	BTV0032A	122	1411	1532	?	0	N	?	N	0.88	after 1547 LS2 group
BAT6018 frame QUSP	BTV0034A<br>BTV0034B	25<br>59			?<br>?	0<br>0	N<br>N	?<br>?	N<br>N	2.16<br>2.91	Undated
BAT6425 Transom plank outer layer QUSP	BTV0035A	65	1392	1456	?	0	N	?	N	2.08	after 1466 ungrouped
BAT6429 Transom plank outer layer QUSP	BTV0036A	66	1503	1568	?	0	N	?	N	1.80	after 1583 LS1 group
BAT6428 Transom plank inner layer QUSP	BTV0037A	171	1408	1578	?	0	N	?	N	0.87	after 1587 B3 group
BAT6428C Transom plank outer layer QUSP	BTV0038A	23			?	0	N	?	N	4.37	Undated
BAT6295 Transom knee QUSP	BTV0039A	26			?	0	N	?	N	3.09	Undated
BAT6229 lodging knee lower deck QUSP	BTV0040_9	128	1431	1558	G	0	N	?	H1	1.72	after 1574 ungrouped
BAT6444 Fashion piece QUSP	BTV0041A	42			?	0	N	?	N	4.74	Undated
BAT6304 Frame timber QUSP	BTV0042A	57			?	0	N	?	N	2.67	Undated
BAT6309 Frame timber QUSP	BTV0043A	64			?	0	N	?	N	1.28	Undated
BAT6340 Frame timber QUSP	BTV0044A	50			?	0	N	?	N	2.29	Undated
BAT6356 Upper fashion piece QUSP	BTV0045A	143	1448	1590	?	0	N	?	N	1.53	after 1605 LS1 group
BAT6220C cleat at transom knee QUSP	BTV0047A	80	1525	1604	G	0	N	?	N	1.22	after 1613 B3 group

*Conversion: R = radial split plank, T = tangential plank, W = whole timber, S = squared whole timber, H = half timber, Q = quarter timber, O = other conversion, Pith: C = centre, V = less than 5 rings, F = 5–10 rings, G = greater than 10 rings.
