# Supplementary material for: Batavia shipwreck timbers reveal a key to Dutch success in 17th-century world trade
Source: PLoS One. 2021 Oct 29;16(10):e0259391. doi: 10.1371/journal.pone.0259391 (PMC8555829; doi:10.1371/journal.pone.0259391)
Supplement: S2 Table — (PDF) [file pone.0259391.s002.pdf]

S2 Table. **Timbers originating from the same tree**. N: number of rings; Pith: C = centre, V = less than 5 rings, F = 5–10 rings, G = greater than 10 rings; SW, number of sapwood rings in the sample. MRW, mean ring width (mm).

Dendro-code	Sample code and species	N	start yr.	end yr.	pith	SW	bark?	extra end	MRW	Interpretation / felling
BAT6086&6391	BAT6086 BAT6391 / QUSP	125	1468	1592	G	0	N	N	1.22	after 1602
BAT6178B&C	BAT6178B&C gunport lid / QUSP	120	1455	1574	?	0	U	N	1.42	after 1590
BAT6213&12a	BAT6213 BAT6389 hull planks inner / QUSP	103	1434	1536	G	0	N	N	1.18	after 1545
BTV00054&66	BAT6069 BAT6169 same tree QUSP	184	1419	1602	?	0	U	N	0.93	Undated
