# Supplementary material for: Batavia shipwreck timbers reveal a key to Dutch success in 17th-century world trade
Source: PLoS One. 2021 Oct 29;16(10):e0259391. doi: 10.1371/journal.pone.0259391 (PMC8555829; doi:10.1371/journal.pone.0259391)
Supplement: S3 Table — (PDF) [file pone.0259391.s003.pdf]

S3 Table. Object chronologies [1]. N: number of rings.

Dendro-code	Object chronology / Nr timbers/ Species	N	start yr.	end yr.
BTV0M001_B3	Baltic 3 group 20 timbers QUSP	275	1342	1616
BTV0M004_LU	Lübeck group 5 timbers QUSP	191	1407	1597
BTV0M002_LS1	LS1 group 10 timbers QUSP	184	1419	1602
BTV0M003_LS2	LS2 group 4 timbers QUSP	135	1398	1532

Reference

1. Daly A, Domínguez-Delmás M, van Duivenvoorde W. Four historical tree-ring chronologies and underlying data derived from dendrochronological research on *Batavia* shipwreck timbers [data set]; 2021. Available from: https://doi.org/10.5281/zenodo.4732901
