# Supplementary material for: Batavia shipwreck timbers reveal a key to Dutch success in 17th-century world trade
Source: PLoS One. 2021 Oct 29;16(10):e0259391. doi: 10.1371/journal.pone.0259391 (PMC8555829; doi:10.1371/journal.pone.0259391)
Supplement: S1 Appendix — (PDF) [file pone.0259391.s004.pdf]

S1 Appendix. Sampling history. The first *Batavia* samples analysed by dendrochronology in 2007 corresponded to five oak (*Quercus* subg. *Quercus*) hull planks and two pine (*Pinus sylvestris*) sheathing plank that had been treated with polyethylene glycol (PEG) after recovery and kept stored in boxes at the Western Australian Shipwrecks Museum. Two of the hull planks (BAT6068A and BAT6068B) were in fact fragments of the same plank, as it was subsequently verified by the dendrochronological results. Samples 5 cm thick were sawn off the timbers and sent to the Ring Foundation in The Netherlands. Standard dendroarchaeological procedures were used for the preparation and research of the samples [1]. To allow the clear visualisation of tree-ring boundaries, two radii were cleaned on the transverse surface of each sample from the inner to the outermost ring. Those samples were measured with a TimeTable measuring device (VIAS, University of Vienna) coupled with the PAST4 software [2] and were compared with a set of reference chronologies for central and northern Europe. For calculating the correlation between the tree-ring series produced, Student's *t*-test [3], adapted for dendrochronology by [4] (hereafter referred to as TBP), was used to identify cross-matching positions. Crossdating provided a date for four of the oak samples, with estimated *post quem* dates ranging between 1534 and 1625, and a possible provenance in the south-eastern Baltic [5]. In 2012, another set of PEG-treated samples (three oak and four pine planks) that had also been kept in boxes was researched. These samples were prepared in the same way for dendrochronological analysis at the laboratories of the Western Australian Shipwrecks Museum in Fremantle. This time, tree rings were recorded in a sequence of overlapping photographs with an automatic camera on macro-lens mode, and ring-widths were subsequently measured at the Ring Foundation using the CooRecorder&CDendro software package [6]. Crossdating was carried out with PAST4 software. None of the samples could then be dated.

In 2015, the sampling strategy targeted different portions of the shipwreck on display at the Western Australian Shipwrecks Museum, so that the results could be representative of the whole wreck. Since the ship had been excavated in separate sections over four years in the 1970s, and those sections had been subsequently mounted for display on top of a metal structure at the museum, we explored the possibility to remove one section from the reconstructed hull to gain access to the cross-sections of the ship's timbers and measure the tree rings. This idea proved however to be an enormous task that would require an inappropriate use of time and resources and was therefore discarded. A different strategy was then adopted, using a Haglöf increment borer (5 mm diameter and 40 cm long) to extract cores from the planks and framing timbers. This sampling method presented several challenges. To gain the most tree-rings in the core, it was necessary to angle the borer perpendicular to the rings, and to core as deep as the material will allow, so that many rings were extracted. The grain of the timbers in the exhibition is very difficult to see, as the PEG preserving them obscures the surface of the wood. For the planks, it was possible to see the layout of the wood grain by inspecting the less well-preserved plank ends, but the position and angle of the core was restricted by the proximity of neighbouring planks, often hindering access. The wood grain was readily visible on some framing timbers, but others were cored without it being possible to see the grain prior to coring, which had varying success. Despite the challenge, cores from 18 timbers were suitable for measurement in 2015. These samples were cleaned on the transverse section with razor blades and measured using a measuring stage developed by Ian Tyers (Sheffield) using a Heidenhain linear encoder; the analysis utilized Tyers' software DENDRO [7] considering also the TBP as crossdating parameter. In this analysis three timber groups were identified, represented by 15 from the new analysis and supplemented with three series from Hanraets, making a total of 18 dated samples [8].

In 2017, a plan for more extensive coring was made, this time using a 7 mm diameter dry-wood borer (Rinntech) powered by a heavy-duty Bosch GBM 13-2 RE 13mm 550W Variable-Speed drill. We attempted to extract a maximum of the available tree rings as possible, from the outermost preserved ones in towards the pith of the original tree. No sapwood was identified on any sample. Sampling PEG-conserved timber with this equipment proved both successful and efficient, and we collected another 91 samples from 74 timbers (including two timbers, BAT6333 and BAT6014 previously sampled in 2015). The same preparation and measuring and crossdating procedures as in 2007 and 2015 were employed. Fifty-three timbers were dated [9]. There are 55 undated samples from 48 timbers. Four samples were not measured, either because they contained too few tree rings, or they were too fragmentary. Again, three main groups of timbers were identified (**Fig A**)

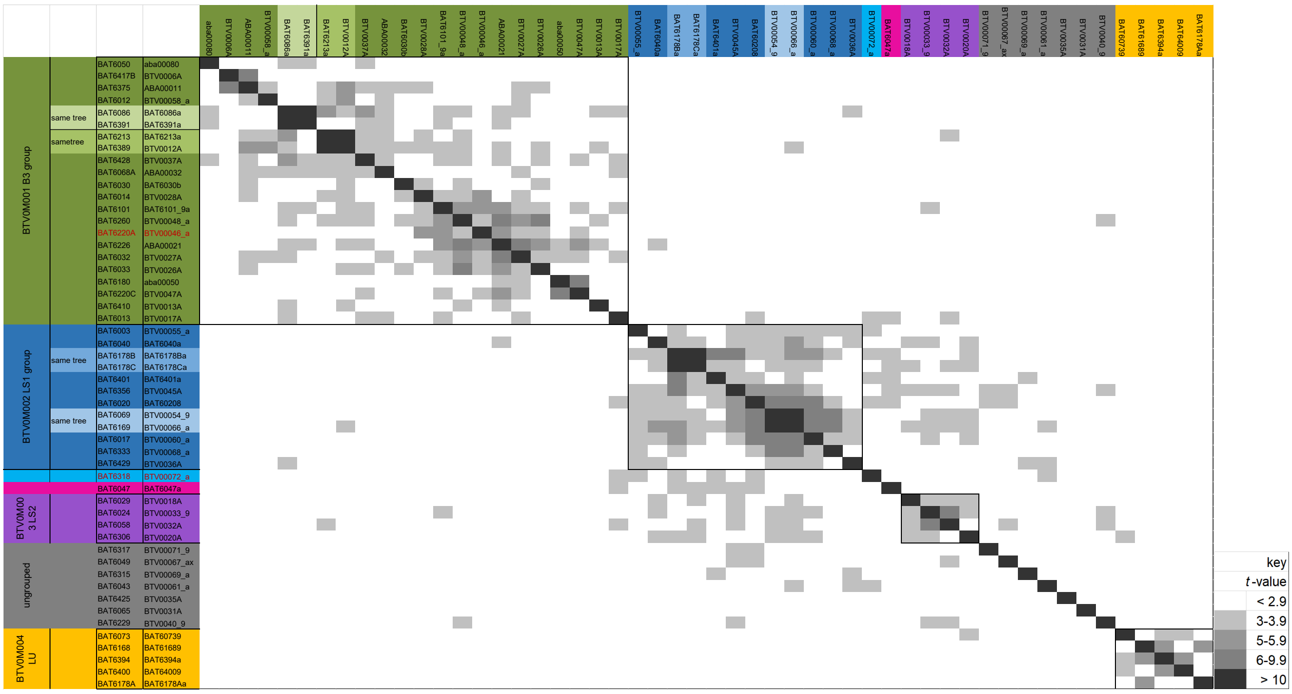

Fig A. Matrix internal correlation. The matrix shows statistical matches (TBP values, see key) between each tree-ring curve with each other, at their cross-dated position. Colour labels correspond with the provenances of the four major groups indicated in Fig 2 of the main text.

References

1. Baillie MGL. Tree-ring dating and archaeology. 1st ed. Chicago: University of Chicago Press; 1982.
2. Knibbe B. PAST4—Personal Analysis System for Treering Research. SCIEM; 2009.
3. Student. The probable error of a mean. Biometrika [Internet]. 1908 Jul 6;6(1):1-25. Available from: http://www.jstor.org/stable/2331554
4. Baillie MGL, Pilcher JR. A simple crossdating program for tree-ring research. Tree-Ring Bull. 1973;33: 7-14.
5. Hanraets E. Report numbers 2007002 and 2007014. Amersfoort; 2007.
6. Larsson L-A. Cybis CooRecorder—Image Coordinate Recording Program & CDendro—Cybis Dendro Dating Program. Cybis Elektronik & Data AB; 2017.

7. Tyers IG. Dendro for Windows. Program Guide. Sheffield; 1997.

8. Daly A. Dendrochronological analysis of timbers from *Batavia*, a Dutch ship, wrecked off the Australian coast. Copenhagen; 2016.

9. Daly A, Domínguez-Delmás M. Dendrochronological analysis of additional timbers from *Batavia*, a Dutch ship, wrecked off the Australian coast. Copenhagen; 2017.
