# Supplementary material for: Batavia shipwreck timbers reveal a key to Dutch success in 17th-century world trade
Source: PLoS One. 2021 Oct 29;16(10):e0259391. doi: 10.1371/journal.pone.0259391 (PMC8555829; doi:10.1371/journal.pone.0259391)
Supplement: S2 Appendix — (PDF) [file pone.0259391.s005.pdf]

S2 Appendix. *Batavia* timbers – forests of origin and selection of trees

While inspecting the shipwreck timbers we noticed that some seemed quite different in terms of tree-growth rate. For dendrochronology, suitable samples should ideally containing at least 80 rings. However, several of the hull planks and (bulky) framing timbers had fewer rings. As a general rule, trees growing in a more open landscape do not need to compete for light, and can grow fast in girth, producing wider rings [1]. In contrast, trees in a dense forest, competing with adjacent trees, tend to grow more slender and produce narrower annual rings. Therefore, the growth rate of the trees can inform about the relative forest cover at the site where the trees grew [2–5]. Plotting in a scattergram the number of rings against mean ring width of the samples analysed and differentiating them according to the provenance groups identified, we can observe the growth rates per region to understand whether the trees for specific timber elements were selected in different areas based on their growth rate. However, in the case of *Batavia*, it must be noted that both pith and sapwood are absent on the samples, so a timber with fewer rings does not necessarily originate from a young tree. More likely, it is a fragment of the stem. Similarly, a timber with high mean ring width (MRW) and low number of rings could be a timber or plank made from a part close to the pith of the tree. Consequently, inferences made from these observations must be cautious.

That said, a clear result from observing the growth patterns and the number of rings in the analysed timbers (Fig A) is that most of the 45 undated samples contained few rings (<60) and the rings were very wide (typically, the wider they are, the fewer there are in a core of course). Given that more than half of these undated timbers are planks (60%) processed tangentially from the parental tree, this result only indicates that the samples were taken at shallow angles (hence the low number of rings) and that the planks likely correspond to the inner part of the tree, closer to the pith, where tree rings are generally wider. Therefore, no inferences can be made about the type of forest cover where the trees grew. However, the rest of the undated timbers are framing elements. Even when the pith and bark are absent in the samples, their wide and few rings suggest that they originate from relatively young trees that have grown fast in a more open landscape. Regarding the dated samples, it is noteworthy that most of them have tree growth rates ranging from 0.75 to 1.75 mm per year, regardless of the number of rings or their origin. The Baltic sourced trees used for the planks are not necessarily slower grown than the Lower Saxony timbers of the frames. Thus, while part of the framing material used for *Batavia* represents usage of younger trees with faster growth (undated framing elements), the rest seems rather homogeneous in terms of growth rates and age of the trees (which were at least between 100 and 200 years old).

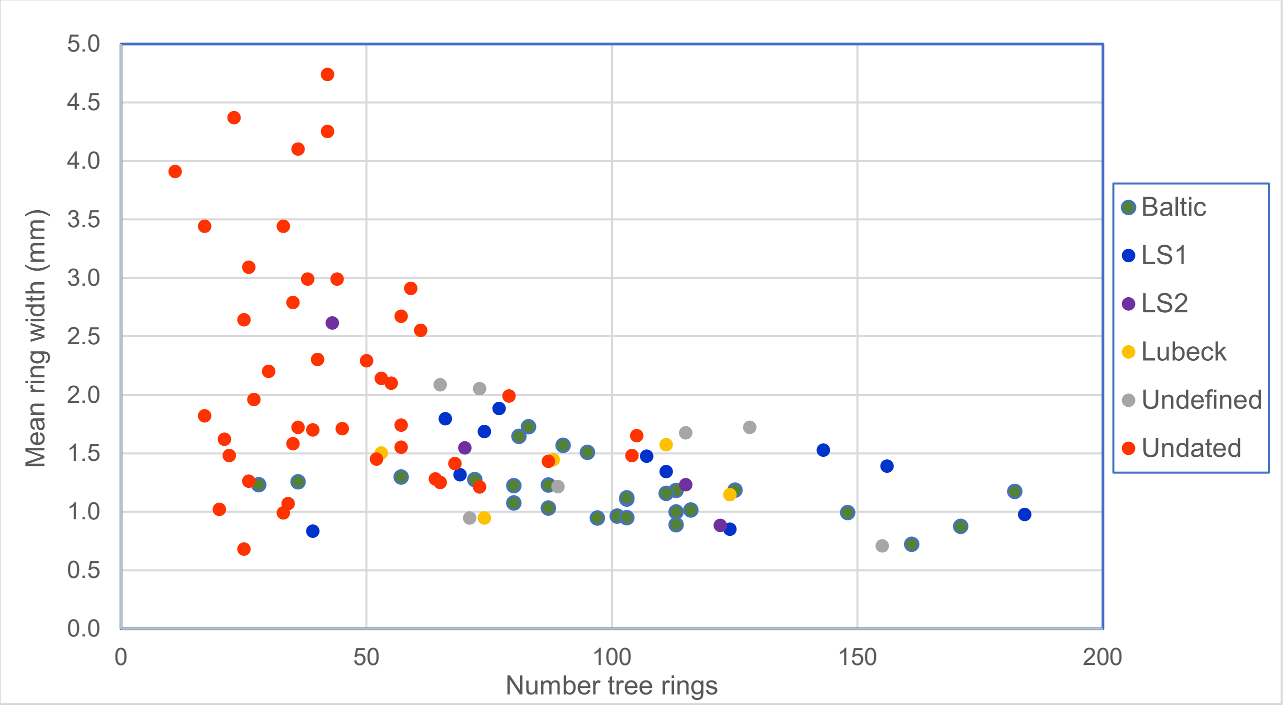

Fig A. *Batavia* timbers tree growth rate expressed as mean ring width. Graph showing the relationship between the mean ring width to the number of rings in each of the samples analysed.

References

1. Schweingruber FH. Tree Rings and Environment. Dendroecology. Berne: Paul Haupt Verlag; 1996.
2. von Arbin S, Daly A. The Mollö cog re-examined and re-evaluated. Int J Naut Archaeol. 2012;41(2):372-389. doi: 10.1111/j.1095-9270.2012.00343.x.
3. Daly A. The Karschau ship, Schleswig-Holstein: dendrochronological results and timber provenance. Int J Naut Archaeol. 2007;36(1): 155-166. doi: 10.1111/j.1095-9270.2006.00103.x.
4. Daly A. Timber-regionality and temporality in Northern Europe's shipbuilding resource. In: Gawronski J, van Holk A, Schokkenbroek J, editors. Ships and maritime landscapes: Proceedings of the thirteenth International Symposium on Boat and Ship Archaeology (ISBSA), Amsterdam 2012. Barkhuis; 2017. pp. 334-338.
5. Domínguez-Delmás M, Rich S, Daly A, Nayling N, Haneca K. Selecting and sampling shipwreck timbers for dendrochronological research: practical guidance. Int J Naut Archaeol. 2019;48(1): 231-244. doi: 10.1111/1095-9270.12329.
